# Time to adequate weight gain and predictors among low-birth-weight preterm neonates at Neonatal Intensive Care Unit of hospitals in Bahir-Dar

**DOI:** 10.1038/s41598-024-66856-7

**Published:** 2024-07-25

**Authors:** Dagnew Tigabu, Hailemariam Gezie, Fekadie Dagnew Baye, Shiferaw Birhanu, Hailemariam Mekonnen Workie

**Affiliations:** 1 Department of Pediatric and Child Health Nursing, College of Health Science, Woldia University, Woldia, Ethiopia; 2 Department of Emergency and Critical Care Nursing, College of Health Science, Wollo University, Dessie, Ethiopia; 3 Department of Pediatric and Child Health Nursing, College of Health Science, Debre Tabor University, Debre Tabor, Ethiopia; 4 Department of Pediatric and Child Health Nursing, College of Medicine and Health Science, Bahir Dar University, Bahir Dar, Ethiopia

**Keywords:** Adequate weight gain, Low birth weight, Preterm neonates, Neonatal intensive care unit, Health care, Medical research

## Abstract

Weight gain in low birth-weight babies remains a challenge to the management of the neonatal period in low and middle-income countries like Ethiopia. Therefore, this study aimed to determine the time to adequate weight gain and its predictors among low-birth-weight preterm neonates admitted to neonatal intensive care unit of public hospitals in Bahir Dar City. An institution-based retrospective follow-up study was conducted from March 4 to April 3, 2023, using three years of data. About 344 low-birth-weight preterm babies were recruited and followed up until 28 days of age. Model goodness-of-fit was checked by Cox Snell residuals test. The Cox-Proportional Hazards Model was used to assess predictors of weight gain with a statistically significant level of *P*-value < 0.05. The median weight gain time was 15 days with an overall incidence density rate of 6.3 per 100 person-day of observation (95% CI 0.055, 0.071). Absence of medical problems of mothers (AHR: 1.63, 95% CI 1.015, 4.614), spontaneous vaginal mode of delivery (AHR: 1.53, 95% CI 1.028, 2.593), and long duration of labor (AHR: 3.18, 95% CI 1.579, 6.413) were significant predictors. The time of adequate weight gain was long. Early detection and management of significant predictors is recommended.

## Introduction

Preterm birth (PTB) refers to the delivery of a baby that occurs before the completion of the 37th week of gestation^1^. Preterm birth occurs between the 28th and 37th weeks of pregnancy. Preterm babies can be early preterm or late preterm. Early preterm babies are born before 34 weeks of gestation, and late preterm babies are born between 34 and 36 weeks of gestation^2^.

The World Health Organization (WHO) defined PTB as a birth that occurs before 259 days following the start of the previous regular menstrual cycle or at a gestational age of < 37 weeks. PTBs account for a significant portion of newborn morbidity and mortality and affect 15 million babies worldwide each year^3^. The prevalence of PTB is rising and continues to be a global concern in both developing and industrialized countries, despite major advancements in the care of preterm newborns^3^.

According to the WHO, low-birth-weight preterm neonates (LBWPTN) are those who are born before 37 complete weeks of pregnancy and weigh less than 2500 g^4^. Weight gain is the amount of weight gained by a newborn in grams per kilogram per day. Weight gain in LBWPTNs is characterized by an initial physiological weight loss of about 7 to 10 percent of birth weight in the first weeks of life, followed by weight gain between the 10th and 21st days of life^5^.

A baby's weight gain depends on intrauterine growth restriction, the amount of early weight loss, and the attention with which practitioners work to achieve the pattern of weight gain depicted by growth charts. The severity of illness is also a significant growth factor, though this has decreased over time in preterm babies^6^.

The development curve indicates that by 36 weeks of gestation, 90% of VLBW neonates fall below the 10th percentile due to inadequate weight gain, which is less than 15 g/kg/day and is a sign of growth delay^5^. A prospective cohort study conducted in Ethiopia revealed that there was a 29% mortality rate for preterm neonates admitted to NICUs, and the majority of survivors (86.2%) had growth restrictions when they were discharged from the medical facility^7^.

Weight increase in preterm neonates is used as a benchmark for hospital discharge as well as a growth indicator^8^. Weight gain occurs during the critical postnatal period in preterm infants and is associated with improved cognitive development^9^.

The causes of inadequate weight gain are not well understood in developing countries, particularly in Ethiopia. In Ethiopia, there is no study conducted on time to adequate weight gain of preterm neonates and its predictors. Therefore, this study aimed to assess the time to adequate weight gain and predictors among LBWPTN admitted at the NICU of public hospitals in Bahir Dar City, Ethiopia.

## Methods and materials

### Study design, area and period

An institution-based retrospective follow-up study was conducted at the NICU of public hospitals in Bahir Dar City from March 4, 2023, to April 3, 2023, by reviewing medical records of LBWPTNs admitted to the NICU from July 08, 2019, to June 07, 2022. The study was conducted at Tibebe Ghion Comprehensive Specialized Hospital (TGCSH), Felege Hiwot Comprehensive Specialized Hospital (FHCSH), and Addis Alem General Hospital (AAGH). The NICUs receive prematurely born babies in the hospitals and those referred from other health institutions. The units are divided into three areas which are the preterm babies’ unit, the term babies’ unit, and the Kangaroo Mother Care unit.

### Population selection and participation

The source populations were medical records of all LBWPTNs admitted to the NICU of Bahir Dar City public hospitals. Medical records of LBWPTNs that were admitted to NICUs from July 08, 2019, to June 07, 2022, and fulfilled the inclusion criteria were included as the study population. Medical records of those LBWPTNs with incomplete daily weight records and those who had major congenital anomalies that interfered with feeding were excluded from this study.

### Sample size determination, sampling technique, and procedure

Double population proportion formula using Epi-info version 7.2.3 was used to calculate the sample size by considering independent predictors of weight gain from previous studies^10,11^, 95% CI, 80% of power, 1:1 ratio of exposed to non-exposed groups. Finally, the sample size was 347 for the study. This sample was allocated proportionally to each study hospital. There are three public hospitals in Bahir Dar city and all were included in this study. Proportional allocation of the sample size was done for each hospital based on the average number of admissions. Firstly, all preterm neonates’ card numbers were obtained from the NICU registration logbook. The total number of premature neonates hospitalized across three hospitals was 2647. All medical record numbers of LBWPTNs admitted to NICUs of the three hospitals were listed as a sample frame. Therefore, the sample sizes drawn from TGCSH, FHCSH, and AAGH were 137, 168, and 42 respectively. Finally, the study subjects for each hospital were selected by a simple random sampling technique.

### Data collection tool, procedure, and quality control

The data were collected using a data extraction checklist adapted from previous literature. The checklist contained four parts; socio-demographic predictors of mothers; socio-demographic predictors of neonates; maternal predictors, and neonatal predictors for weight gain. The data extraction tool was pretested on 5% of the sample size two weeks before the actual data collection period at FHCSH by reviewing the medical records of LBWPTNs admitted to the NICU before July 8, 2019. Moreover, two-day practical training was given to the data collectors and the supervisor on data collection methods, the objective of the study, and how to review weight gain follow-up from medical records. Data were collected by three trained nurses working in the selected hospitals and supervised by a master-level pediatric and child health nurse practitioner.

### Study variables and their measurements

The outcome variable was time to adequate weight gain among LBWPTNs and this was measured as an event and censored.

#### Event

Weight gain of about 15-20gm/kg/day of LBWPTNs within 28 days of life^10,10^.

#### Censored

Other than the events such as against medical advice, referred to other health institution, lost follow-up, death before the event, admission after one week of age (left-censored), and not weight gain < 15 g/kg/d until 28 days of life^10,10^.

#### Follow-up time

At the time of admission (must be before one week) up to 28 days^10^.

#### Preterm

Those newborns delivered before 37 completed weeks of gestation^10^.

#### Low-birth-weight

Weight of neonates less than 2500 g during delivery.

### Data processing and analysis

The data were cleaned, coded, and entered into Epi data version 4.6. Then exported to STATA version 17 statistical software for the analysis. Descriptive statistics were computed depending on the nature of the variables, and results were presented as texts, graphs, and tables. The outcome of each participant was dichotomized into an event and censored. Incidence Density Rate (IDR) was calculated for the entire study period. Kaplan Meir (KM) was used to estimate median survival time and a KM plot with a log-rank test was used to compare the grouped variables. Model goodness-of-fit was checked by Cox Snell residuals (then the cumulative hazard versus cox-Snell residuals curve closely followed the 45-degree line) and assumptions were checked by using the Schoenfeld residual test and those variables with a *p*-value > 0.05 and graphically not cross each other, were entered into the model. Multicollinearity was also checked and variance inflation factor (VIF < 5) and pairwise correlation < 0.7 were accepted. For each independent predictor, bi-variable Cox proportional hazard regression was performed and variables with a *p*-vale < 0.25 were included in multivariable Cox proportional hazard regression. Lastly, variables at a 95% confidence interval with a *p*-value < 0.05 were considered statistically significant predictors of weight gain. Additionally, missing values were identified in five variables (age of mothers, residence of mothers, medical problem of mothers, Place of delivery, and PROM), and the percentage ranged from 2.1 to 6.2%. The pattern of missing was not random, and mean imputations were implemented to fill the missed values.

### Ethics approval and consent to participate

Ethical approval was obtained from the Institutional Review Board (IRB) of the College of Medicine and Health Sciences, Bahir Dar University with reference number 12788/24, and Ethiopian Public Health Institute Amhara region branch (APHI) with reference number  to conduct the study. Ethics approval and consent to participate in this study were confirmed by the IRB of College of Medicine and Health Sciences, Bahir Dar University, and APHI confirming that all the methods were performed according to the rules and regulations of the IRB, Hospitals, and by the 1964 Helsinki Declaration. Informed consent was impracticable to this study. This study was done by the Helsinki Declaration which stated that “medical research using identifiable human material or data, physicians must normally seek consent for collection, analysis, and storage and/or reuse. There may be conditions where obtaining informed consent from the participants would be impossible or impracticable and threaten the validity of the research. In this instance, the research may be done after considerations and approval of the IRB”. The ethical approvals were submitted to study area hospitals. Letters of permission were also obtained from the hospitals. Finally, permission letters were submitted to the hospitals’ Neonatal Department card rooms. Thus, the informed consent of the participants was waived by the IRB of the College of Medicine and Health Sciences, Bahir Dar University and APHI. The collected data were kept anonymous and confidentiality was maintained.

## Results

### Sociodemographic characteristics of mothers

From the total 344 LBWPTNs’ medical record reviews, 103 (29.9%) mothers were between the ages of 31 and 35 years, and 175 (50.9%) mothers lived in urban areas.

### Previous maternal obstetric condition

Only 39 (11.4%) of the mothers had a history of preterm delivery. Similarly, 32 (9.4%), 35 (10.2%), and 34 (9.9%) mothers had a history of stillbirth, experienced pregnancy-related problems during their pregnancy time, and had a history of birth complications respectively. 313 (91%) mothers had ANC follow-up during the pregnancy of the current baby (Table 1).

**Table 1:** Maternal obstetric conditions of mothers of low-birth-weight preterm neonates admitted to NICU of public hospitals in Bahir Dar city, Ethiopia, 2023.

Variable	Category	Adequate weight gain		Frequency with %	IDR/100	PDO
		Event	Censors			
Previous history of preterm delivery	No	195 (56.7%)	110 (32%)	305 (88.7%)	6.2	3138
	Yes	25 (7.3%)	14 (4.1%)	39 (11.4%)	6.8	366
Previous History of stillbirth	No	203 (59%)	109 (31.7%)	312 (90.7%)	6.2	3235
	Yes	17 (4.9%)	15 (4.5%)	32 (9.4%)	116.3	269
History of any birth complication	No	199 (57.8%)	111 (32.3%)	310 (90.1%)	6.2	3206
	Yes	21 (6.1%)	13 (3.8%)	34 (9.9%)	7.0	298
ANC follow-up	Yes	205 (59.6%)	108 (31.4%)	313 (91%)	6.2	3266
	No	15 (4.4%)	16 (4.7%)	31 (9.1%)	6.3	238
Any medical problem	No	199 (57.9%)	113 (32.8%)	312 (90.7%)	6.4	3128
	Yes	21 (6.2%)	11 (3.2%)	32 (9.4%)	5.6	376
Pregnancy related problem	No	201 (58.4%)	108 (31.4%)	309 (89.8%)	6.4	3149
	Yes	19 (5.5%)	16 (4.7%)	35 (10.2%)	5.3	355

*IDR* Incidence Density Rate, *PDO* Person-Day Observation, *ANC* Ante Natal Care.

### Labor and delivery condition of the indexed mothers

About one-quarter of the mothers [84 (24.4%)] gave birth in their homes. Regarding mode of delivery, 297 (86.3%) mothers gave birth through spontaneous vaginal delivery (SVD). And also 292 (84.9%) mothers delivered within 12 h. Most of the mothers, 317 (92.1%) feed their children within one hour of delivery (Table 2).

**Table 2:** Neonatal conditions of low-birth-weight preterm neonates admitted to the NICU of public hospitals in Bahir Dar city, northwest Ethiopia, 2023.

Variable	Category	Adequate weight gain		Frequency with %	IDR/100	PDO
		Event	Censors			
Place of delivery	Health institution	208 (60.5%)	52 (15.1%)	260 (75.6%)	6.2	3321
	Home	12 (3.5%)	72 (20.9%)	84 (24.4%)	6.5	183
Mode of delivery	SVD	182 (52.9%)	115 (33.4%)	297 (86.3%)	6.1	2958
	C/S	38 (11.1%)	9 (2.6%)	47 (13.7%)	6.9	546
Duration of labor	≤ 12 h	211 (61.3%)	81 (23.6%)	292 (84.9%)	5.4	3390
	> 12 h	9 (2.6%)	43 (12.5%)	52 (15.1%)	7.9	114
PROM	No	206 (39.9%)	107 (31.1%)	313 (91%)	6.3	3278
	Yes	14 (4.1%)	17 (4.9%)	31 (9%)	6.2	226
Initiation of feeding	Within one hours	203 (59%)	114 (33.1%)	317 (92.1%)	6.2	3259
	After one hours	17 (4.9%)	10 (2.9%)	27 (7.8%)	6.9	245

*IDR* Incidence Density Rate, *PDO* Person-Day Observation, *PROM* premature Rupture of Membrane, *SVD* Spontaneous Vaginal Delivery, *C/S* Cesarean Section.

### Neonatal condition

Among the entire LBWPTNs included in this study, 216 (62.8%) were females, 317 (92.1%) had normal Apgar scores in the first and fifth minutes, and 59 (17.2%) were early preterm. About 312 (90.7%) of neonates were feeding through breast milk and most of the neonates 322 (93.6%) were fed every three hours. The majority of the LBWPTNs (73%) stayed for > 7 days in the NICUs of the hospitals. About one-third (34.9%) of the neonates had medical problems during the follow-up time. The overall IDR/100 were 10.8 and 2623 total follow up time in day (Table 3).

**Table 3:** Neonatal conditions of low-birth-weight preterm neonates admitted to the NICU of public hospitals in Bahir Dar city, northwest Ethiopia, 2023.

Variable	Category	Adequate weight gain		Frequency with %	IDR/100	PDO
		Event	Censors			
Sex of neonate	Male	85 (24.7%)	43 (12.5%)	128 (37.2%)	6.2	1359
	Female	135 (39.3%)	81 (23.5%)	216 (62.8%)	6.3	2145
Gestational age	Late Preterm	198 (57.6%)	87 (25.2%)	285 (82.8%)	6.3	3139
	Early preterm	22 (6.4%)	37 (10.8%)	59 (17.2%)	6.0	365
APGAR score	≤ 6 (Abnormal)	9 (2.6%)	18 (5.2%)	27 (7.8%)	6.2	145
	≥ 7 (Normal)	211 (61.3%)	106 (30.8%)	317 (92.1%)	6.2	3359
Initiation methods of feeding	Tube	101 (29.4%)	61 (17.7%)	162 (47.1%)	6.4	1562
	Breast Feeding	119 (34.6%)	63 (18.3%)	182 (52.9%)	6.1	1942
Frequency of feeding	Every two hour	8 (2.3%)	14 (4.1%)	22 (6.4%)	5.2	153
	Every three hour	212 (61.6%)	110 (32%)	322 (93.6%)	6.3	3351
Type of milk	Breast	201 (58.4%)	111 (32.3%)	312 (90.7%)	6.2	3242
	Mixed	19 (5.5%)	13 (3.8%)	32 (9.3%)	7.2	262
Neonates have any medical problem	No	156 (45.3%)	68 (19.8%)	224 (65.1%)	6.3	2479
	Yes	64 (18.6%)	56 (16.3%)	120 (34.9%)	6.2	1025
Duration of hospital stays	≤ 7 days	49 (14.2%)	44 (12.8%)	93 (27%)	6.1	806
	> 7 days	171 (49.7%)	80 (23.3%)	251 (73%)	6.3	2698

*IDR* Incidence Density Rate, *PDO* Person-Day Observation, *APGAR* Appearance Pulse Grimace, Activity Respiration.

### Time to adequate weight gain of low-birth-weight preterm neonates

**Figure 1:**
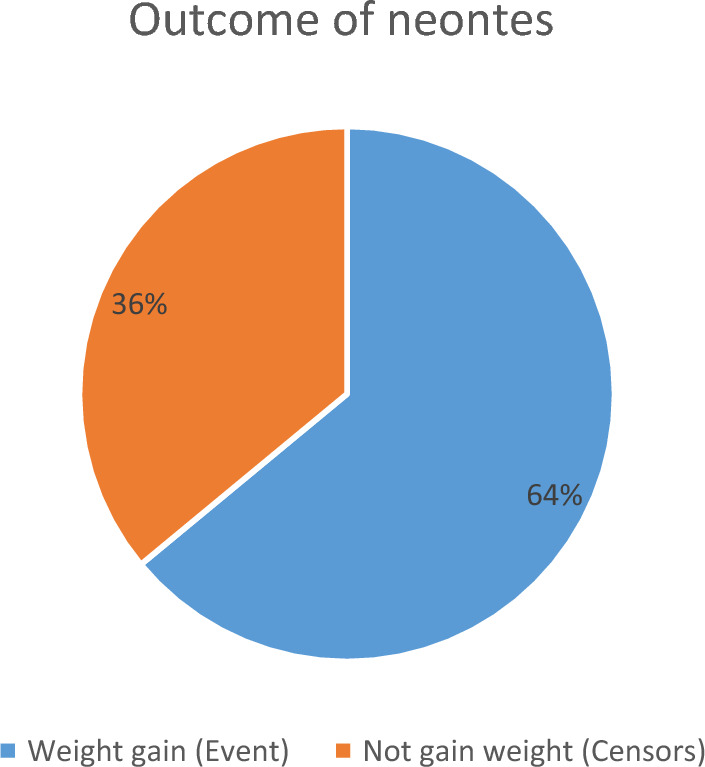
The outcome (weight gain) status of low-birth-weight preterm neonates admitted to the NICU of public hospitals in Bahir Dar city during the follow-up period, northwest Ethiopia, 2023.

During the follow-up period, average weight loss on 7.3% (95% CI 3–19%) LBWPTNs before the occurrence of weight gain. The study was followed for a minimum of 10 days to a maximum of 28 days which gave a total of 3504 person-day of observation. The Incidence rate of weight gain was 6.3 per 100 Person–day observation. Within the follow-up period, the median time of adequate weight gain was 15 days of age, which was recorded among 220 (64%) (95% CI 0.587, 0.688) preterm neonates with an average weight gain of 10.5 g/kg/day (Fig. 1).

### Kaplan–Meier survival function or weight gain probability

**Figure 2:**
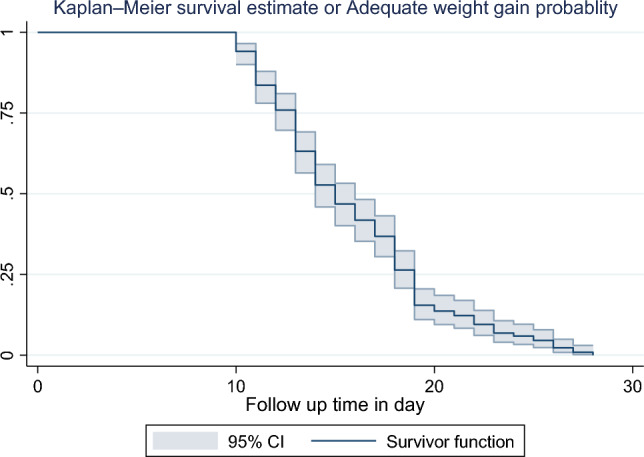
The Kaplan–Meier survival estimates of time to adequate weight gain and predictors among admit low-birth-weight preterm neonate admitted to the NICU of public hospitals in Bahir Dar-city, northwest Ethiopia, 2023.

A total of 344 LBWPTNs were followed for a minimum of 10 days and a maximum of 28 days, which gave 3504 person-day observation, and from these, 220 (64%) had adequate weight gain at the end of the study period. The median time for weight gain was 15 days. Hence, the overall incidence density rate (IDR) during the cohort was 6.3 per 100 person-day of observation (95% CI 0.055, 0.071) (Fig. 2).

### Log-rank test result comparison on different categorical variables

The equality of the survival curves for various categorical explanatory variables was examined using the log-rank test. The test statistics demonstrated that the survival function for various categorical explanatory variables differed significantly from one another. Mothers' medical problems (whether they were present or not), the mode of delivery (SVD vs C/S), and the length of labor (≤ 12 vs > 12 h) were among the explanatory variables that significantly differed. Mothers with medical problems were contrasted with mothers without such problems in this study. The p-value of 0.0383 indicated that this difference was statistically significant. The same applies to labor hours with fewer than twelve hours being preferred to more than twelve hours with a p-value of 0.004, this difference met the criteria for statistical significance. Additionally, SVD delivery differs from C/S in terms of mode of delivery with a p-value of 0.0063, and this difference was statistically significant.

### Proportional hazard assumption

For each variable, a test of the scaled Schoenfeld residuals proportional hazard assumption and the overall global test were conducted. The overall global test had a *p*-value of 0.8748, and each variable's *p*-value was > 0.05. This indicates that the assumption was satisfied (Table 4).

**Table 4:** scaled Schoenfeld residuals proportional hazard assumption test for each variable and overall global test among low-birth-weight preterm neonates admitted to the NICU of public hospitals in Bahir Dar city, northwest Ethiopia, 2023.

Predictors	rho	chi^2^	df	P. Value
Residence	− 0.03152	0.28	1	0.5976
Pregnancy related problem	0.00947	0.03	1	0.8742
Birth complication	− 0.03709	0.39	1	0.531
PROM	0.04826	0.67	1	0.4136
Birth weight	− 0.00996	0.03	1	0.8675
Methods of feeding	− 0.05003	0.71	1	0.3981
Global test	**2.44**	**6**	**0.8748**	

*Rho* Correlation Cofficient, *Chi*^*2*^ Chi Square, *df* Degree of Freedom , *PROM* Premature Rapture Of Memebrane.

### Testing the model goodness of fitness

**Figure 3:**
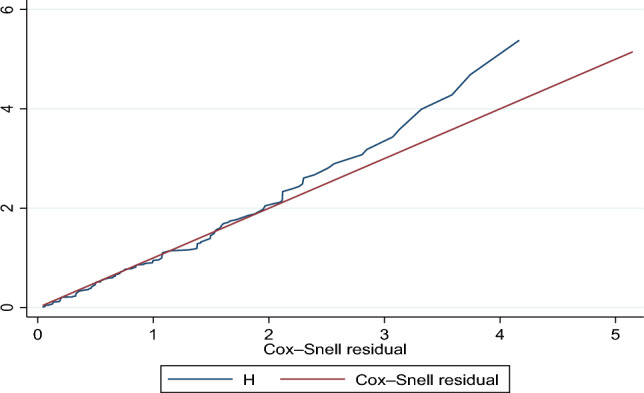
Cox Snell residual test to the goodness of fit of Cox proportional hazard model among low-birth-weight preterm neonates admitted to the NICU of public hospitals in Bahir Dar city, northwest Ethiopia, 2023.

The cox-Snell residual test was used to determine the cox-proportional hazard regression model's goodness of fit. It was calculated based on the Kaplan–Meier estimated failure function to assess the overall suitability of the fitted model. We can see from the graph below that the model matches the data since the cumulative hazard versus cox-Snell residuals curve closely follows the 45-degree line (rho = Correlation Cofficient, Chi2 = Chi Square, df = Degree of Freedom, PROM = Premature Rapture Of Memebrane Fig. 3).

### Predictors for adequate weight gain

Variables with *p*-vale < 0.25 in the bi-variable Cox regression were included in the multivariable Cox proportional hazard regression model. Then absence of medical problems of mothers, spontaneous mode of delivery, and duration of labor < 12 h were significant predictors for weight gain among LBWPTNs in the multivariable Cox regression analysis at 95% CI and *p*-value < 0.05 with AHR. LBWPTNs whose mothers had no medical problems during pregnancy were 1.63 times more likely to gain adequate weight as compared with neonates whose mothers had medical problems during pregnancy (AHR: 1.63, 95% CI 1.028, 2.593). Similarly, LBWPTNs delivered with SVD were 1.53 times more likely to gain adequate weight as compared to neonates delivered by C/S (AHR: 1.53, 95% CI 1.050, 2.254). Additionally, LBWPTNs delivered within 12 h of the duration of labor were 3 times more likely to gain adequate weight as compared to those preterm neonates delivered after 12 h of duration of labor (AHR 3.18, 95% CI 1.579, 6.413) (Table 5).

**Table 5:** Bi-variable and multivariable Cox regression analysis of predictors of adequate weight gain among low-birth-weight preterm neonates admitted to the NICU of public hospitals in Bahir Dar city, northwest Ethiopia, 2023.

Predictors	Category	Adequate weight gain		CHR (95% CI)	AHR (95% CI)
		Event	Censors		
Mode of delivery	C/S	38	9	1.0	1.0
	SVD	182	115	0.37 (0.098, 0.849)	1.53 (1.050, 2.254) *
Medical problem of mother	Yes	21	11	1.0	1.0
	No	199	113	0.92 (0.141, 0.998)	1.63 (1.028, 2.593) *
Birth complication	No	21	13	1.0	1.0
	Yes	199	111	1.01 (1.001, 1.974)	1.54 (0.966, 2.463)
Frequency of feeding	Every three hours	8	14	1.0	1.0
	Every two hours	212	110	3.37 (2.252, 3.957)	0.54 (0.261, 1.136)
Initiation of feeding	Within one hour	203	114	1.04 (1.002, 1.277)	0.67 (0.399, 0.926)
	After one our	17	10	1.0	1.0
Methods of feeding	Tube	101	61	0.87 (0.615, 0.960)	1.08 (1.012, 1.428)
	Breast	119	63	1.0	1.0
History of preterm	No	195	110	0.99 (0.453, 1.056)	0.7 (0.458, 0.920)
	Yes	25	14	1.0	1.0
Duration of labor	≤ 12 h	211	81	2.44 (1.210, 2.811)	3.18 (1.579, 6.413) *
	> 12 Hours	9	43	1.0	1.0
Types of milk	Breast	201	111	1.23 (1.034, 1.894)	0.68 (0.410, 0.850)
	Mixed	19	13	1.0	1.0

*CHR* Crude Hazard Ratio, *AHR* Adjusted Hazard Ratio, *CI* confidence Interval, *SVD* Spontaneous Vaginal Delivery, *C/S* Cesarean Section.

* = Significant at *p* < 0.05.

## Discussion

This study aimed to determine the time to adequate weight gain and its predictors among LBWPTNs receiving care at Bahir Dar City Public Hospitals and it revealed an incidence rate for weight gain was 6.3 per 100 person-days observation. At the end of the observation period, 64% of LBWPTNs had adequate weight gain within the first 28 days of life. This finding was higher than studies conducted in Kenya (27.4%)^13^, and Tanzania (13.4%)^10^. This discrepancy could be because of variations in participants, the current study participants were low-birth-weight preterm neonates whereas the Kenyan and Tanzanian studies enrolled all preterm neonates, sample size might be another reason in which the previous studies had a smaller sample. The Tanzania study was a prospective cohort and the Kenyan study was cross-sectional which might also be a factor for the difference. There are also differences in the general population of the three countries such as feeding practices, lifestyle of mothers, and environmental factors.

The median time for adequate weight gain according to this observation was 15 days. This result was in line with studies conducted in Uganda Kampala^11^ and Turin University Italy^14^. This might be due to similarities in the study participants in which the current study, the Uganda and Italy studies included LBWPTNs. However, it was lower than a study conducted in four countries (Guinea‐Bissau, Nepal, Pakistan, and Uganda)^15^, and another Italian study^16^. This difference may be because the majority of these findings were made in late-preterm neonates with a larger sample size. Earlier preterm neonates do not have good eating tolerance and their GI is not also mature enough to facilitate weight gain and this leads to slower weight gain among early preterm than late preterm neonates.

In this study, the average weight gain was 10.7 gm/kg/day. This finding was slightly higher than studies conducted in Tanzania (5.07 gm/kg/day)^10^ and Southern India (9.5gm/kg/day)^17^. This might be due to variations in sample size, study design, and participants in which most participants of the above studies were early preterm, and early preterm neonates are exposed to feeding intolerance which further leads to slower weight gain. On the other hand, this average weight gain was lower than studies done in South Africa (13.2 gm/kg/d)^12^, Ohio State University USA (11.1gm/kg/d)^5^, Mexico (17 gm/kg/d)^18^, and India (15.18 gm/kg/d)^19^. Even this average weight gain is lower than the WHO standard of the average daily weight gain of preterm infants (15 to 20 gm/kg/d). The possible reasons for the discrepancy might be variations in sample size in which the above studies included smaller samples and the study design of the above studies was cross-sectional.

According to this study, LBWPTNs from mothers who did not have any medical problems during pregnancy were 1.63 times more likely to get adequate weight gain as compared to neonates from mothers who had medical problems during pregnancy. This finding was supported by studies done in Tanzania Kilimanjaro^20^, and South Africa^12^. The possible justification is that neonates who were delivered from mothers who had no medical problem may have adequate feeding, early breast feeding initiation, and be free from congenital problems and this, in turn, lead to early weight gain.

Low-Birth-Weight Preterm Neonates delivered with SVD were 1.53 times more likely to gain adequate weight as compared with those delivered by C/S. This finding was in line with the findings of London^21^ and Iran^22^. This might be because neonates delivered by C/S may not have early skin-to-skin contact with their mothers and early breastfeeding may not be possible as the mothers are in the post-operative phase with the effects of anesthesia and this affects the early weight gain of the neonates. Additionally, neonates delivered by C/S lose more weight after delivery and gain weight more slowly than vaginally delivered babies because they are more hydrated when they are born because the mother received intravenous fluids before the procedure. The other possible justification might be that neonates who were delivered by vaginal delivery had exposure to normal floras, which are important for gut development and help to train the immune system and produce vitamin K. As immunity and absorption increases weight gain also increases.

Moreover, this study revealed that LBWPTNs delivered within 12 h of duration of labor were three times more likely to gain adequate weight as compared to those low-birth-weight preterm neonates delivered after 12 h of duration of labor. The possible reason might be that prolonged labor can cause consequences such as infection or sepsis, birth trauma, and electrolyte disturbances. For this reason, the neonates may not gain adequate weight early.

## Conclusion and recommendation

The findings of this study revealed that only 64% of LBWPTNs gain adequate weight and the time to gain adequate weight was long. The absence of medical problems of mothers, SVD, and duration of labor ≤ 12 h were predictors for adequate weight gain among LBWPTNs admitted to the NICU. Therefore, the Amhara Regional Health Bureau, Bahir Dar City Administration Health Department, hospitals, and the health care workers shall work for early detection and management of the identified predictors and shorten the time to gain adequate weight.

## Data Availability

All the data are presented in the manuscript and raw data are available from the corresponding author of any reasonable request.

## Abbreviations

IDR: Incidence Density Rate
PTB: Preterm Birth (PTB)
WHO: World Health Organization
LBWPTN: Low Birth Weight Preterm Neonates
VLBW: Very Low Birth Weight
NICU: Neonatal Intensive Care Unit
FHCSH: Felege Hiwot Comprehensive Specialized Hospital
TGCSH: Tibebe Gion comprehensive Specialized Hospital
AAGH: Adiss Alem General Hospital
KM: Kaplan Meir ()
VIF: Variance Inflation Factor
PROM: Premature Rapture of Membrane
ANC: Antenatal Care
SVD: Spontaneous vaginal delivery
C/S: Caesarean Section
APHI: Amhara public health institute
IRB: Institutional Review Board
AHR: Adjusted Hazard Ratio
